# Interactions between avian viruses and skin in farm birds

**DOI:** 10.1186/s13567-024-01310-0

**Published:** 2024-04-26

**Authors:** Laurent Souci, Caroline Denesvre

**Affiliations:** Laboratoire de Biologie des Virus Aviaires, UMR1282 ISP, INRAE Centre Val-de-Loire, 37380 Nouzilly, France

**Keywords:** Avian viruses, skin tropism, feathers, keratinocytes, in vitro skin models, chicken, duck

## Abstract

This article reviews the avian viruses that infect the skin of domestic farm birds of primary economic importance: chicken, duck, turkey, and goose. Many avian viruses (e.g., poxviruses, herpesviruses, Influenza viruses, retroviruses) leading to pathologies infect the skin and the appendages of these birds. Some of these viruses (e.g., Marek’s disease virus, avian influenza viruses) have had and/or still have a devasting impact on the poultry economy. The skin tropism of these viruses is key to the pathology and virus life cycle, in particular for virus entry, shedding, and/or transmission. In addition, for some emergent arboviruses, such as flaviviruses, the skin is often the entry gate of the virus after mosquito bites, whether or not the host develops symptoms (e.g., West Nile virus). Various avian skin models, from primary cells to three-dimensional models, are currently available to better understand virus-skin interactions (such as replication, pathogenesis, cell response, and co-infection). These models may be key to finding solutions to prevent or halt viral infection in poultry.

## Introduction to avian skin and integuments

Since the chicken is the most studied bird in terms of pathology and anatomy, as well as the third animal model in biomedical research [1], chicken skin will be used as a reference herein. Important differences between chicken and duck, goose, or turkey skins will be mentioned at the end of this section.

The chicken skin or tegument consists of the skin itself, invaginated glands, and protruded appendages, such as feathers and scales. It ensures diverse roles: (i) physical protection against external aggressors including pathogens, (ii) regulation of thermal, hygrometric, and chemical parameters, (iii) waterproofing, providing a permeability barrier to prevent water loss, (iv) matting and locomotion [2].

### Organization of the chicken skin

**Figure 1:**
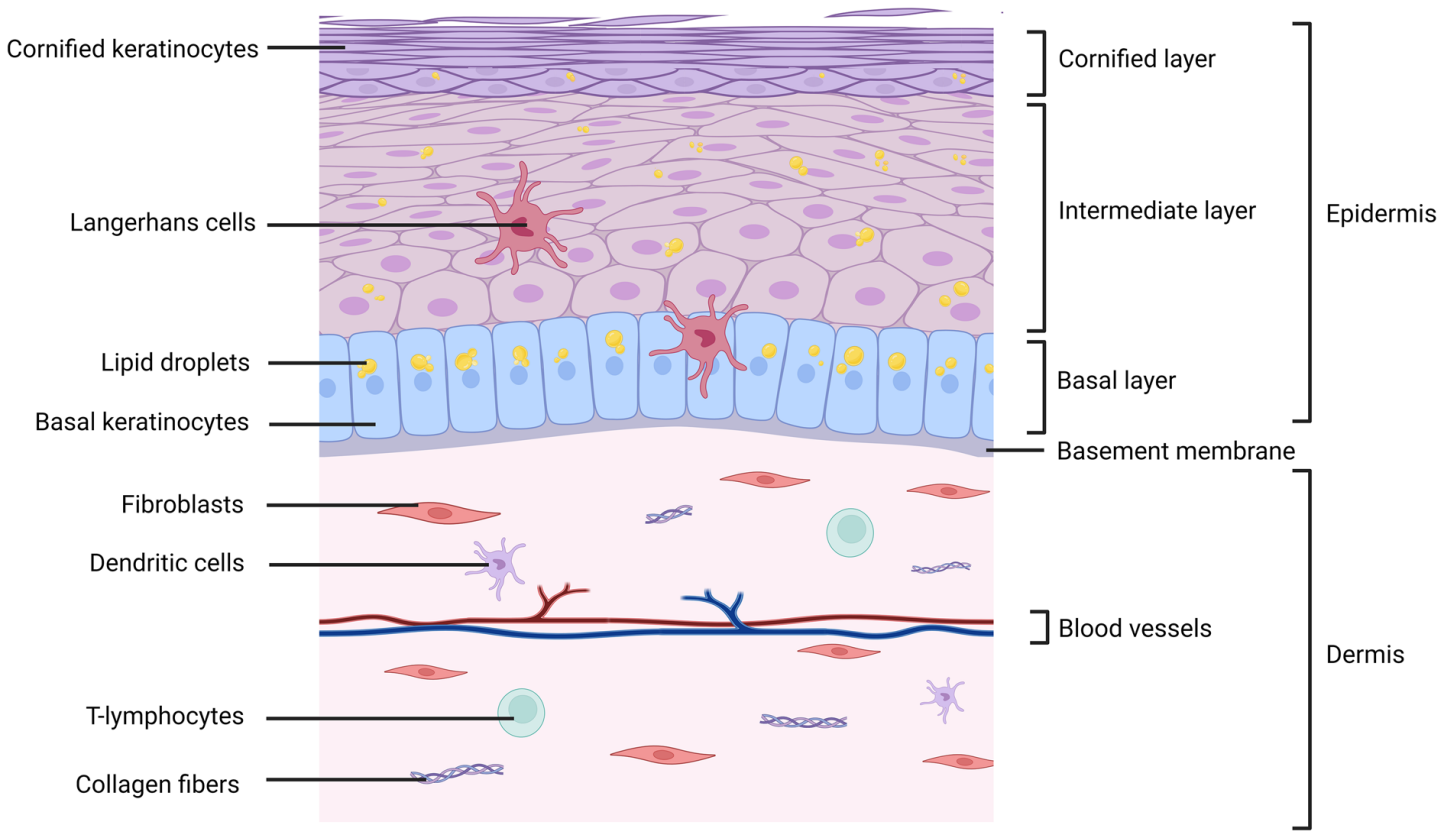
**Structure of the avian skin.** Skin is constituted by an epidermis and a dermis separated by the basement membrane. Three layers constitute bird’s epidermis: the basal layer made of undifferentiated basal keratinocytes, the intermediate layer and the cornified layer made of cornified keratinocytes that are fully differentiated. Keratinocytes are rich in lipids and/or lipid droplets. The dermis is a fibrous vascularized structure mainly composed of fibroblasts and of matrix components such as collagen fibers. By analogy to mammals, it is probable that dendritic cells and T-lymphocytes also reside in the dermis. Langerhans cells are present in basal but also intermediate layers of the epidermis. This figure was illustrated by using BioRender.com (Agreement number: XJ26EG35BS).

Like in mammals, chicken skin consists of a dermis covered by an epidermis, both separated by a basement membrane [3] (Figure 1). The dermis is composed of different cell types (fibroblasts, endothelial cells, and immune cells) and is rich in dense irregular connective tissue. The epidermis is a stratified squamous epithelium, containing mostly keratinocytes. The epidermis of chicken is composed of three layers, from the deepest to the most superficial: (i) the basal layer, (ii) the intermediate layer, and (iii) the cornified layer. Keratinocytes proliferate in the basal layer and then differentiate and stratify gradually into suprabasal layers through a process called terminal differentiation [4]. This is accompanied with cornification, a programmed cell death (as reviewed by Eckhart [5]). This results in flat dead keratinocytes (called corneocytes) which cover the surface of the epidermis in contact with the air or water [5]. In unfeathered skin, the maintenance of the epidermal homeostasis is secured by a constant shedding of corneocytes balanced by a cell replacement with differentiating keratinocytes. The new keratinocytes arise from a pool of epidermal stem cells located in the basal layer [5, 6]. Chicken skin presents several major differences with mammal skin. The chicken epidermis is thinner, given the absence of the granular layer [7]. Chicken keratinocytes accumulate intracellular lipid droplets [8, 9], a feature that protects against dehydration in absence of sebaceous glands. Lastly, avian epidermis exhibits feathers, instead of hair in mammals.

### Hard and soft skin appendages in chicken

**Figure 2:**
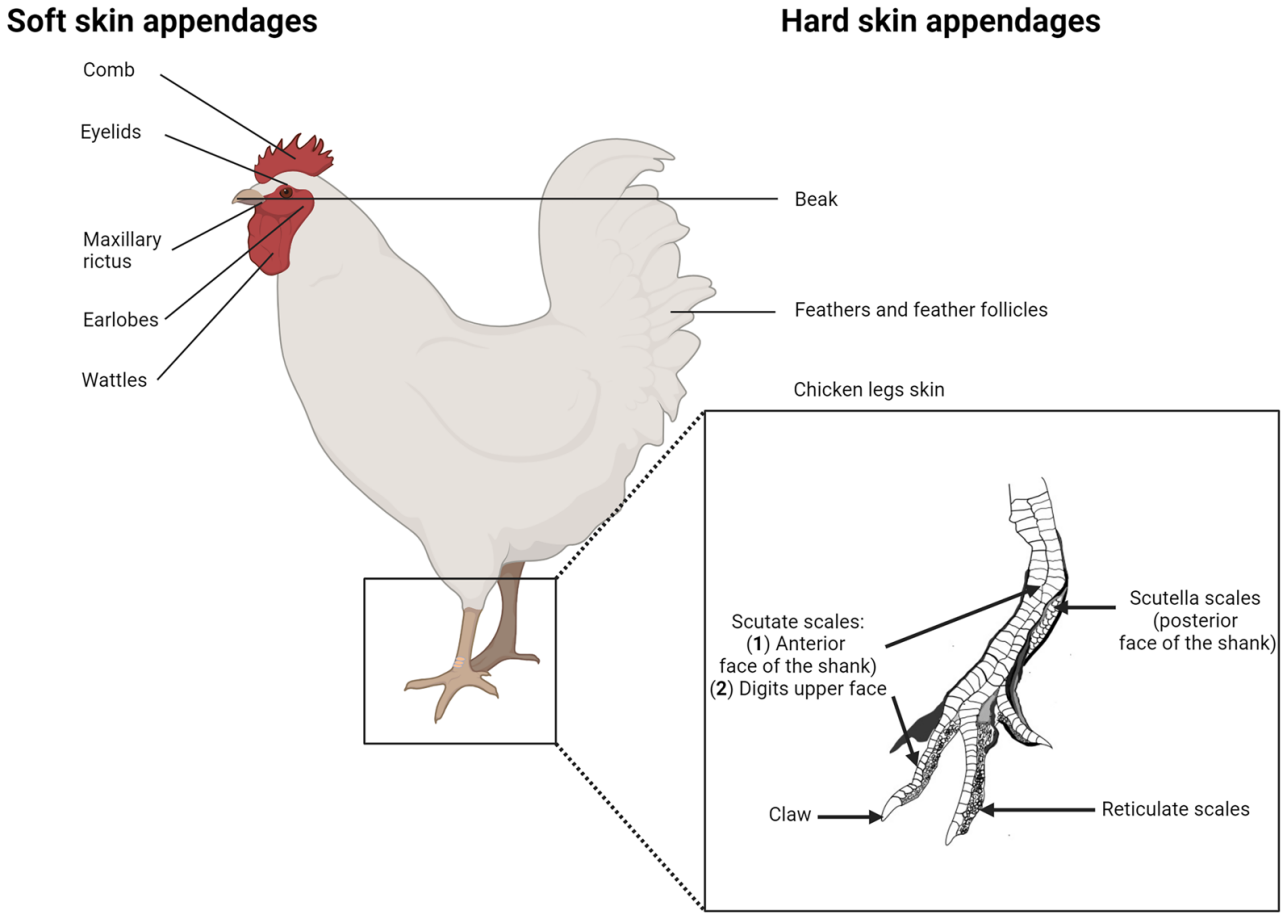
**Hard and soft skin appendages in birds/chicken.** On the left panel, soft skin appendages including comb, eyelids, maxillary rictus, earlobes and wattles are shown. On the right panel, hard skin appendages including beak, feathers, feather follicles and chicken legs skin are shown with a special focus onto legs scales. Legs possess different hard cornified elements: claw, scutate scales (large and distally overlapping scales) onto digits and anterior face of the shank, scutella scales (smaller and proximally overlapping scales) on the posterior face of the shank and finally reticulate scales (smaller) that recover the remainder of foot surface and are non-overlapping. This figure was illustrated by using BioRender.com (Agreement number: GC26L9TWNH) and Inkscape software.

About a century ago (1883), Jeffries stated that “the epidermis of birds possesses a much greater variety of appendages than that of any other vertebrate group” [3]. Birds possess several hard skin appendages (feathers, scales, claws, and beak) composed mainly of alpha-keratins (as in all vertebrates), but also of corneous beta-proteins (also referred in the past as beta-keratins) unique to birds and reptiles [10–12]. Corneocytes from hard skin appendages are characterized by a heavily cross-linked cytoskeleton, a rigid and chemically resistant envelope, and very tight cell-to-cell connections that impede cells desquamation [3, 5]. Feathers are the most abundant cutaneous appendages, covering the major surface of the body [13]. Each feather arises from a feather follicle (FF), a self-renewing mini-organ invaginated into the skin, through a series of complex events [14]. Scales are highly keratinized extensions of the cornified layer and ensure both physical protection and water loss prevention [15]. Three types of scales exist in chicken: (i) reticulate scales from footpads (ii) scutate scales from the dorsal part of the toe, and (iii) scutella scales that are smaller and positioned lateral to scutate scales [15, 16] (Figure 2). Differences occur between avian species: land birds (e.g. chicken, turkey) have hard cornified feet skin scales (podotheca), while water birds (e.g. duck) have softer ones [17, 18]. Beak and claw structures consist of a hard cornified epidermis (forming a sheath) that cover the bones of the jaw and feet, respectively [11, 19].

Several integumentary outgrowths also named soft skin appendages are present on the head and upper neck of chicken [3, 17] including the maxillary rictus, the comb, the earlobes, and the wattles, all localized in the glabrous region [2] (Figure 2). Such structures are composed of soft epidermis [11, 20]. Eyelids are also considered as soft skin appendages. They ensure cornea protection and are constituted by an upper and a lower lid made of a loose fold of skin (unfeathered or with delicate feathers, depending on the bird species) covering eyes globes [21]. A third eyelid, the nictitating membrane, is a protective fold of skin [17].

### Immune component of the skin

The immune component of the skin and appendages of birds was poorly studied yet, including in chicken. It is likely that dendritic cells (Langerhans cells and dermal dendritic cells), macrophages, and T lymphocytes are present, as shown in healthy human and mouse skin (for review see [22, 23]). In chicken skin, the Langerhans cell is the best characterized immune cell. Langerhans cells are present in chicken epidermis of featherless skin, and its number has been estimated at 2000 per mm^2^ in an 8-week-old chick [24–26]. In contrast to mammals, the presence of intraepithelial lymphocytes was not demonstrated yet. In addition, a role for avian keratinocytes in the immune response is probable, as shown in mammals for example by sensing virus components [22], but this has never been reported for birds. Concerning chicken skin appendages, some immune cell populations were quantified from the pulp of growing feathers and wattles by flow cytometry and immunohistochemistry [27]. In feather pulp sensu stricto, Erf et al. estimated by immunochemistry less than 1% CD4-positive cells, less than 2% CD8-positive cells and about 7% MHCII-positive cells. Using transgenic 8 day-old-chicks (“MacRed” chickens) in which monocytes and tissue macrophages expressed a red fluorescent reporter protein, Balic et al. reported numerous macrophagic cells in feather pulp [26].

## Viruses infecting avian skin and/or skin appendages

This section will focus on viruses infecting farm birds of primary importance: chicken, duck, turkey, and goose. A few pet or wild bird viruses will be also mentioned when important. A large number of avian viruses show a tropism for the skin and its related appendages. These viruses belong to families well known for their tropism for the skin in mammals (e.g. *Poxviridae*, *Herpesviridae*, and *Papillomaviridae*), to families containing arboviruses (arthropod-borne disease) entering the host through arthropod bites (e.g*.* Flaviviruses), and to families that do not usually replicate in the skin of mammals (e.g. *Retroviridae*, Influenza viruses) (Table 1). In the sections below, viruses will be referred to by their common name, but the taxonomic name will also be mentioned. All virus infections reviewed in this chapter are more extensively covered in Swayne’s *Disease of Poultry* book [28].

**Table 1:** **Avian viruses with skin-tropism**

Virus family	Virus genus	Virus species including common names	Host species	Disease
DNA				
*Polyomaviridae*	*Polyomavirus*	Goose hemorrhagic polyomavirus (GHPV)	Goose	Haemorrhagic nephritis enteritis
	*Polyomavirus*	Avian polyomavirus (APV)/Budgerigar fledgling disease virus (BFDV)	Parrot	Budgerigar fledgling disease also called Avian polyomavirus infection, non budgerigar polyoma infection
*Papillomaviridae*	*Papillomavirus*	Fringalla Coelebs papillomavirus 1 (FcPV1), *Pygoscelis adeliae* Papillomavirus 1, PaPV-1, psittacus erithtacus, papillomavirus 1 (PePV-1) …	Parrot, wild birds	Cutaneous lesions, tumors
*Parvoviridae*	*Dependoparvovirus*	Anseriform dependoparvovirus 1	Muscovy duck, mule, Peking duck and goose	Derzsy's disease
	*Parvovirus*	Goose Parvovirus (GPV)	Goose, duck (muscovy)	Derzsy's disease
*Poxviridae*	*Avipoxvirus*	Fowlpox (FPV)	Chicken	Dry pox or wet pox
		Turkeypox virus (TPV)	Turkey	
		Goosepox (HGP)	Goose	
*Herpesviridae*	*Mardivirus*	Gallid herpesvirus type 2 (GaHV2)/Marek disease virus (MDV)	Chicken	Marek Disease (MD)
		Mealagrid herpesvirus (MeHV1)/Turkey herpesvirus (HVT)	Chicken, turkey	Not pathogenic
		Gallid herpesvirus type 3 (GaHV3)	Chicken	Not pathogenic
	*Iltovirus*	Gallid herpesvirus type 1 (GaHV1)/Infectious laryngotracheitis virus (ILTV)	Chicken	Infectious laryngotracheitis
*Circoviridae and Anelloviridae*	*Circovirus*	Goose circovirus (GoCV)	Goose	Immunosuppression, feathers disorders
		Duck circovirus (DuCV)	Duck	Immunosuppression, feathers disorders
		Psittacine beak and feather disease virus (BFDV)	Parrot, Psittacine birds	Psittacine Beak and Feather Disease
	*Gyrovirus*	Chicken anemia virus (CAV)	Chicken	Chicken infectious anemia disease
RNA				
*Retroviridae*	*Alpharetrovirus*	Avian leukosis virus (ALV)	chicken	Immunosuppression, leukaemia-like proliferative diseases
	*Gammaretrovirus*	Reticuloendotheliosis virus (REV)	chicken, turkey, duck, goose and quail	Immunosuppression, cell lymphomas, feathers disorders, “Nakanuke” disease
*Orthomyxoviridae*	*Alphainfluenzavirus*	Avian Influenza type A virus	Duck (domestic, waterfowl), goose, chicken, turkey	Avian Flu
		Highly pathogenic viruses (HPAIV) (H5N8, H5N1…)		
		Low pathogenic viruses (LPAIV) (of all H and N subtypes)		
*Flaviviridae*	*Flavivirus*	West Nile Virus (WNV)	Birds	Encephalitis and other neurological disorders
		Usutu virus (USUV)	Birds	Severe neurological disorders
		Duck Tembusu virus (DTMUV)	Duck	Egg drop syndrome, neurological disorders

### Poxviruses

Poxviruses infecting birds belong to the *Avipoxvirus* genus in the *Poxviridae* family and cause fowlpox disease, a common disease with economic consequences in poultry (reviewed in [29–31]). They are large double-stranded DNA viruses, with an unconventional envelope. In farms, avipoxviruses (APV) are more prevalent in tropical and subtropical countries than in northern countries [32]. Three APV species can be involved: fowlpox virus, turkeypox virus, and goosepox virus [33]. All of them have a strong tropism for skin and are known to induce cutaneous lesions. Transmission generally occurs directly by stitching and scratching or most commonly following insect bites. Indirect transmission between birds may also occur by aerosol inhalation or by ingesting infected scabs/dust [31].

The fowlpox virus being the most important APV in poultry, it will be taken as an example in this section. The fowlpox virus infects chicken and turkey [34], causing fowlpox disease, for which two clinical forms are described: a cutaneous form (the most common) and a diphtheritic form. The cutaneous form or “dry fowlpox” is a slow-spreading skin disease with low mortality. It is characterized by hyperplasia of the epidermis (acanthosis), ballooning of keratinocytes, and the formation of large eosinophilic intracytoplasmic inclusions containing virion particles [35]. The nodular or crusty lesions are mainly observed in unfeathered areas of skin, but also in the comb, wattles, and eyelids of chicken [36] and turkey [34]. Hard skin appendages such as beaks, claws, and feet skin may eventually present lesions. Feather lesions are atypical, but occasionally described [37, 38]. The recovery rate for dry fowlpox is high.

The diphtheritic form usually occurs after ingestion or inhalation of the virus and is more severe, with up to 15% of mortality [32]. This form is characterized by lesions located in diverse mucosa (mouth, esophagus, larynx, or trachea) [35, 36]. Vaccines are available for fowlpox prevention. Chicken and turkey are vaccinated with a live attenuated strain of fowlpox or pigeonpox virus [39]. The spread of infection being slow, vaccination within the flock can be performed during the early phases of the outbreak [31].

Until now, permissive culture systems used to replicate APV are mostly primary chicken embryonic fibroblasts (CEFs) [36], DF-1 cell line [32], and embryonated chicken eggs inoculated onto their chorioallantoic membranes [36]. Therefore, cell interaction and cell response to infection have been studied mostly in these systems rather than in cell types naturally infected in vivo, such as keratinocytes.

### Herpesviruses

Four avian herpesviruses in the *Herpesviridae* family show a tropism for skin. They all belong to the *Alphaherpesvirus* subfamily, with three from the *Mardivirus* genus (Marek’s disease Virus, Herpesvirus of turkey, Gallid herpesvirus type 3) and one from the *Iltovirus* genus (Infectious laryngo-tracheitis virus). Herpesviruses are large enveloped viruses, with a linear double-stranded DNA genome.

#### Marek’s disease virus

Marek’s disease virus (MDV or Gallid herpesvirus type 2, GaHV2), the prototype of the *Mardivirus* genus, is mostly known for inducing lethal T-cell lymphoma in chicken. This virus was disastrous before vaccines availability in the 1970s. Today, this virus is still found worldwide and economically important for the poultry industry. The infection occurs by inhalation of contaminated dander present in poultry dust. In the first week of infection, the virus infects lymphocytes and the feather follicle epithelium (FFE) [40–43]. Whereas T-lymphocyte infection leads mostly to a latent cycle, FFE infection leads to a lytic cycle and virion production. In 2022, by imaging the whole body of experimentally-infected chickens, we discovered that MDV also infects the skin of the legs covered with scales, the beak, and the base of the claws [43]. Therefore, MDV is capable of infecting and replicating in all hard skin appendages of the chicken but not in soft skin appendages. Infection was detected in small areas of each skin appendage. In the epidermis, MDV replication was limited to the intermediate layer and never observed in the basal layer [43–45]. By electron microscopy, mature virion particles were observed in quantity only in the cytoplasm of keratinocytes from the FFE [46], whereas very few mature particles were present in other cell types in vivo (reviewed in [47, 48]).

MDV replicates persistently in the FFE of non-vaccinated chicks until death [49], but also in the FFE of vaccinated chicks [50]. Therefore, viral replication in the FFE appears not to be controlled by the immune response. Feathers debris (especially of the feather outer sheath) and dander contained in farm dust are considered as the major source of MDV [51, 52] and horizontal transmission between individual chickens [42]. Surprisingly, farm dust remains infectious for months into the environment [53, 54], which is unusual for a herpesvirus. This suggests that the mature enveloped virions are not “free” but are physically protected from degradation by a material that remains to be identified. In addition of being the site of virus shedding the skin can also be the site of small tumors, often diagnosed due to swollen FF with lymphoid aggregates [42].

Until now, MDV is cultivated mostly in primary chicken or duck embryo fibroblasts. There is currently no cell culture system that enables the production of mature virions as seen in FFE in vivo [47, 55]. Even if chicken keratinocytes derived from embryonic stem cells developed in our laboratory [56] or chicken skin explants prepared from unfeathered skin of 18–20-day-old embryos [57] have been able to sustain MDV infection, they did not produce mature virions efficiently. It remains unknown why complete particles maturation is restricted to differentiated keratinocytes of the FFE.

#### Herpesvirus of turkey and Gallid Herpesvirus type 3

The herpesvirus of turkey (HVT or Mealagrid herpesvirus type 1, MeHV1), naturally infects turkeys [58]. As HVT is not pathogenic for the chicken and highly related antigenically to MDV, this virus was initially used to vaccinate chickens against Marek’s disease [59, 60]. Since the 2000s, this virus is widely used as a vaccine for numerous poultry pathogens (reviewed in [61]). After inoculation to chicks, HVT reaches the FFE [62] and persists for months (and up to the chicken lifetime) in this tissue [63]. Although HVT genome is present in dander [64], HVT spreads very poorly between chickens [65].

Like HVT, the Gallid herpesvirus type 3 (GaHV3) is non-pathogenic for the chicken and used to vaccinate against Marek’s disease [60]. GaHV3 also replicates into the FFE [62] and is shed in dander [64]. In contrast to HVT, GaHV3 efficiently spreads between chickens and is probably circulating “silently” among farms. Indeed, we reported the presence of GaHV3 genome on the skin surface of healthy chickens, sampled from a French experimental farm [66], in which GaHV3 vaccine had never been used. The presence of this virus was also suspected in UK flocks, after detection of GaHV3 sequence by qPCR from feather tip material (S. Baigent, personal communication).

HVT and GaHV3 are cultivated on the same cell systems than MDV. Infection of HVT was also reported on chicken skin explants prepared from unfeathered skin of 18- to 20-day-old embryos, although no mature virions were observed by electron microscopy [57].

#### Infectious laryngo-tracheitis virus

The infectious laryngo-tracheitis virus (ILTV or Gallid herpesvirus type 1, GaHV1) causes a respiratory disease in chicken, with various degrees of severity. ILTV replicates predominantly in the epithelial cells of the trachea and conjunctiva of infected chickens, inducing lesions of the mucosa [28]. Clinical symptoms include a dry or crusty ocular discharge around the eyes and the eyelids. Although ILTV does not display a true skin tropism, the ILTV genome was detected in feather material from infected or vaccinated chickens [67, 68]. Even if the ILTV genome can be found in feathers and dust [68, 69], it is unclear if these materials may be source of ILTV transmission.

### Circoviruses and gyroviruses

Circoviruses and Gyroviruses are small icosahedral non-enveloped, circular single-stranded DNA viruses that belong to the family of *Circoviridae* and of *Anelloviridae*, respectively. Circovirus DNA is ambisense, whereas Gyrovirus DNA is negative sense [28]. More than 60 bird species can be infected by such viruses, with immunosuppression as a consequence in most cases. Clinical symptoms related to skin appendages are described below.

#### Goose circovirus

The goose circovirus (GoCV) has been described in farm and wild geese in Europe and Asia [70]. GoCV infection causes immunosuppression with nonspecific clinical symptoms and promotes secondary infection by other pathogens (e.g., goose parvovirus). GoCV can also cause feather disorders, with atrophy or insufficient development of feather follicles [71]. A study conducted with goslings from about 40 Taiwanese farms, showed that infected animals had feather loss at 21–35 days of age and/or broken feathers at 42–60 days of age [72]. In GoCV-positive geese, FFs presented necrosis with inclusion bodies [72].

#### Duck circovirus

Similar to GoCV, the duck circovirus (DuCV) causes immunosuppression and favors secondary infection [73, 74]. All ducks infected with DuCV exhibit feathering disorder, feather loss, and growth retardation [73, 75, 76]. Feather dystrophy with haemorrhagic shafts was observed along the dorsum of ducks [77]. DuCV was detected in farm ducks (Muscovy, Pekin or mule ducks) but also in wild ducks [75, 78]. These data come mainly from naturally occurring or experimental bird infections, given that no cell culture system is currently available for propagation of DuCV [76]. Synergistic effects between DuCV and other viruses are observed: for example, coinfection by DuCV and Goose parvovirus potentiates replication and pathogenicity [79].

#### Psittacine beak and feather disease virus

Although not affecting farm birds, the Psittacine beak and feather disease virus (PBFDV) nicely illustrates the tropism of this virus family for the skin and its appendages. This virus, discovered in 1984, affects most (if not all) Psittaciformes, endangering some species [80]. In its chronic form, the psittacine beak and feather disease is characterized by feather loss and deformed beak and claws [80]. These lesions are caused by epidermal hyperplasia, necrosis of epidermal cells, and hyperkeratosis with an excessive amount of scales. Normal feathers are progressively replaced by dystrophic ones after their molting [81, 82]. Intracytoplasmic inclusion bodies can be observed in feather epithelial cells and macrophages [81]. The virus can be transmitted horizontally from bird to bird through feces, contaminated feather dust, and crop secretions [83], but also vertically from mother to embryos. It appears to persist in the environment, allowing indirect transmission.

#### The chicken anemia virus

The chicken anemia virus (CAV), first isolated from chicken in 1979 in Japan is the agent responsible for the chicken infectious anemia disease, an economically important immunosuppressive poultry disease [84]. CAV is the unique member recognized among *gyrovirus*, a genus that has been reclassified from *Circoviridae* to the new *Anelloviridae* family [85]. Briefly, CAV causes aplastic anemia, immunosuppression, reduced growth, and lymphoid tissue atrophy in young chicken [86]. In chickens older than 3–4 weeks, CAV causes mild subclinical infection due to the transient immunosuppression that can result in secondary infections. In the skin, only occasional/marginal symptoms can be observed such as (i) subcutaneous haemorrhages localised to the wings that may turn blue and break (Blue-wing disease) (mainly in co-infection context) [87], and (ii) skin lesions subsequent to secondary bacterial infection. Feathers constitute one potential source of infection [88]. CAV can be transmitted vertically and horizontally mainly by the faecal-oral route, but sometimes through infected FFE. When newly hatched chickens were experimentally inoculated at oral or ocular mucosal surfaces with CAV extracted from feathers, the virus was detected 7–14 days post-infection [86, 88]. In the same study, infectious CAV was found in the feathers’ shaft, surface, and pulp and was shed from feathers [88].

### Retroviruses

In the *Retrovidae* family, in the *Orthoretrovirinae* sub-family, two avian virus groups have been shown to interact with the skin and feathers: the avian leukosis virus (ALV) from the *Alpharetrovirus* genus (reviewed in [89]) and the reticuloendotheliosis virus (REV) from the *Gammaretrovirus* genus (reviewed in [90]). Retroviruses are enveloped RNA viruses encoding a reverse transcriptase, which generates a DNA provirus that integrates into the host genome.

#### Avian leukosis virus

Avian leukosis viruses (ALV) are often classified in subgroups (currently 11) based on their envelope glycoprotein (e.g. subgroups A, B, C, D, J, and E). The E subgroup contains only endogenous viruses, whereas the subgroups A, B, C, D and J contain exogenous viruses. Natural infection with exogenous ALV occurs in chickens. The virus is shed by the hen into the albumen, contributing to vertical transmission. This mode of transmission is exclusive, except for ALV-J, which is also transmissible horizontally. For all ALV, the progeny of an infected hen is tolerant to the virus and lacks neutralizing antibodies. This tolerance leads to viral persistence and tumorigenesis, resulting in either lymphoid leukosis or myelocytomatosis [89].

ALV can be isolated from many samples of infected chickens [89], including the feather pulp by group specific antigen testing and visualized by transmission electron microscopy (TEM) [91]. In TEM, retroviral particles are visible in the intercellular space of the epidermis of the FF, but also budding from epidermal cell membranes [92].

For ALV-J, retroviral particles have been observed by TEM in various regions of the feather epithelium (epidermal collar, intermediate layer of the feather epidermis), in accordance with the detection of the p27 viral antigen by immunochemistry [93]. The virus has been detected in the feather pulp of infected chickens, usually at higher titers than in plasma or cloacal swabs [94], making the feather a sample of choice for diagnostic. For this reason, the feather pulp is preferentially used to detect ALV-J, notably by PCR [94–96]. Therefore, it has been suspected (but not yet proven) that feathers could be the source of ALV-J in horizontal transmission. Broilers (young chickens) infected in ovo by ALV-J show feather abnormalities in remiges [93] (thinness, increase transparency of the calamus, sparseness of the vane), indicating that virus replication alters feather development.

Endogenous ALV (ev) infection has been detected in feather pulp cells through group-specific antigen detection [97]. Interestingly, in White Leghorn chicken, a dominant sex-linked late-feathering allele (K) was characterized by a slow growth of wings and tail feathers. This mutation was found associated to the integration of ev21 (an ALV-E), suggesting a relationship between ev21 and feather development [98].

#### Reticuloendotheliosis virus

REV natural infection occurs in chickens, turkeys, ducks, geese, and quails. REV is transmitted horizontally by contact or possibly by blood-sucking insect bites, and vertically in chickens and turkeys. Infections with replicative strains induce three types of pathology, which are relatively rare in flocks [90]: runting syndrome, immunosuppression, and cell lymphomas. The runting syndrome may be associated with abnormal development of the feathers [90], named Nakanuke disease, which consists of feathers with barbs adhering to a small portion of the shaft [99, 100], as well as feathers with increased transparency and thinness of the calamus and rachis and loss of proximal barbs [101]. These feather abnormalities were initially reported after injection of one-day-old chicks with a REV isolated as contaminant of HVT-infected cells [99]. They were observed in various contour feathers, but mostly in the flight feathers of the wings [101]. These lesions appear to be due to REV‐induced necrosis of feather‐forming epithelial cells of the developing barb ridges [101]. Viral particles can be observed by TEM in feathers and occasionally in FFs. In feathers, virions are restricted to the epidermal collar, ramogenic zone, and barb ridges, especially in cells of the intermediate layer [101]. Virions are mostly present in interepithelial spaces. No particles were detected in the dermal papilla and the feather pulp [101]. This indicates that REV has a tropism for and a high replication rate in the epithelial cells of the feathers. REV attenuated for its oncogenic properties by passage on cells retains its ability to induce feather abnormalities [102]. Feather abnormalities were also observed when REV was accidentally transmitted to chicks through contaminated vaccines (HVT or fowlpox virus) [100, 103]. Of note, REV sequences were also found inserted in field isolates of fowlpox virus [104], suggesting that the two viruses can co-infect the same cell type, possibly keratinocytes. Experimentally, REV can be transmitted by close contact with infected birds, but not when chickens are separated by wire mesh [90]. This indicates that REV is rapidly inactivated in the environment.

### Influenza viruses type A

Avian influenza type A virus (AIV) are members of the *Orthomyxoviridae* family. They are enveloped viruses, with a segmented negative-sense single-strand RNA genome. The virus classification relies on the two major glycoproteins, the hemagglutinin (H) and the neuraminidase (N) (for example H5N1 or H7N7). AIV are naturally widespread in wild aquatic birds, which are considered as the major reservoir [105], and can naturally infect most farm birds [106]. AIV infections are either asymptomatic in poultry, or can cause avian influenza, a systemic and highly lethal disease. The symptom severity of the disease depends on various parameters such as the virus pathotype, the host (bird species, age, host immunity), co-infection occurrence with other pathogens, and environmental factors (reviewed in [107]).

Highly pathogenic avian influenza viruses (HPAIV) (only of H5 and H7 subtypes) induce severe disease with high mortality and morbidity in chickens and other Galliformes, but variable clinical signs and mortality in ducks [108]. HPAIV can cause outbreaks with considerable economic losses, due either to virus morbidity and mortality in infected flocks, high transmissibility, or strict measures taken to limit virus spread in a geographical area (reviewed in [107]). HPAI viruses spread systemically, when most low pathogenic avian Influenza (LPAI) viruses remain confined to mucosa. If most LPAIV (of all H and N subtypes) induce no or mild clinical symptoms, mostly respiratory, some can induce severe respiratory signs. In addition, LPAIV with H5 or H7 represent a real threat for poultry as they may mutate into HPAIV.

In Galliformes, in acute cases induced by HPAIV, hemorrhages can be observed in many tissues, especially in unfeathered skin and soft appendages [109].

In 2007, for the first time, Yamamoto et al. reported necrotic lesions of the feather epithelium in domestic call ducks experimentally infected by intravenous inoculation with an H5N1 HPAIV strain [110]. In 2-week-old ducks, lesions were observed 3 to 7 days post-infection (dpi), especially in growing feathers, and were distributed from the epidermal collar to the pulp cap of the feather [110]. Focal necrosis was also observed in the epithelium of the beak and of the scaled skin of the legs, suggesting a tropism for most hard skin appendages [110]. Abundant expression of viral antigens was detected in necrotic lesions [110] and infectious virus was isolated from 1 to 7 dpi in skin harboring numerous small feathers, with titers above 10^3^ (determined by the 50% egg infectious dose/g) [110]. Virions were observed in the feather epithelium of infected ducks by TEM [111].

The presence of H5N1 HPAIV in feathers was also reported from naturally infected ducks (Pekin and Muscovy) initially by reverse transcription quantitative PCR, in Vietnamese farms [112]. Abundant antigens were detected by immunochemistry in nearly all feathers of ducks infected by a Vietnamese H5N1 HPAIV, and in all skin tracts [113]. Viral antigens were located in the epidermis of feathers and follicles, with higher amount in feathers [113]. The proportion of feathers positive for viral antigens was lower in the feathers of ducks infected with two other H5N1 of Indonesian clades, suggesting different degree in feathers tropism and/or replication among H5N1 HPAIV. Importantly, H5N1 was detected and isolated from duck feathers with and without clinical signs [111].

Experimentally, nasal inoculation of call ducks with H5N1 confirmed that a natural infection route leads to feather infection [114]. In addition, oral inoculation of call ducks with feathers from a H5N1 infected duck leads to infection, indicating that feathers contain infectious material and are a possible source of horizontal transmission between birds and possibly mammals [114].

The feather tropism is a property shared by HPAIV, H5N1, but also H7N1 [115] and H5N8 [116]. Indeed, viral RNA was detected in growing feathers of naturally infected ducks during the recent outbreaks of clade 2.3.4.4b H5N8 and H5N1 in France. Moreover, in domestic ducks experimentally infected with H5N8, Gaide et al. showed (by examining viral antigen location in growing feathers by immunochemistry at 3 and 5 dpi) that the virus diffuses into the feather from the dermal pulp and the marginal plate of the epithelium to barbs and barbules [117]. This suggests that the virus follows the path of keratinocyte growth and differentiation. By micro-dissecting the infected growing primary feathers, the authors found a moderate viral infectivity in all parts of the feather, but statistically greater infectivity in newly formed barbs/barbules (around 10^5^–10^6^ focus forming unit/mL) compared to the outer sheath (10^2^–10^3^ plaques forming unit/mL) and dermal pulp [117].

The presence of LPAIV in feathers was not reported. This is not surprising because these viruses usually remain in the respiratory and digestive mucosa and do not lead to a systemic infection. For the few LPAIV shown to spread hematogenous, it would be interesting to search for the presence of the virus in feathers. The replication of HPAIV in the feather epithelium is not restricted to ducks and was also reported after natural or experimental infections in other farm birds: chicken [112, 115, 117–119], goose [111, 116], turkey [117], and quail [117]. In chickens infected with H5N1 HPAIV, lesions and viral antigens are mostly found in the dermis of feathers and follicles, in contrast to ducks where they are mostly detected in the feather epithelium [113]. A tropism for the dermis was also observed in H5N8-infected chickens [117]. Therefore, depending on the bird species, the same virus shows a preferential tropism for the dermis or epidermis.

AIV infectivity in feathers persists for a long time. In feathers detached from infected domestic ducks, H5N1 infectivity lasts for 15 days at 20 °C and 160 days at 4 °C [120], and can persist up to 240 days in chicken feathers at 4 °C. Preen oil secreted by the uropygial gland plays a role in H5N1 stability on duck feathers [121].

Due to the persistence of AIV infectivity in feathers and to the higher amount of virus in feather samples than in oropharyngeal and cloacal swab samples at almost all time points post-infection, feathers could be used as reference samples for AIV surveillance and diagnosis [112, 115, 116, 118, 122, 123].

Finally, the presence of AIV in feathers raises the crucial question of the presence of infectious HPAIV in dust and fomites, and the possibility of HPAIV spread through airborne particles on relatively long distances. Recently, histological analysis of dust collected from HPAIV-positive farms revealed a co-staining for viral antigen with a corneous-ß-protein, a feather marker [117] indicating that feather dust contains viral material. Recently, James et al. reported the presence of infectious H5N1 in dust samples collected outside poultry houses during an epizooty in the UK [124]. These data suggest that poultry dust and possibly feathers may play a role in HPAIV transmission. This is an important question that will need to be addressed in the future.

### Flavivirus

Flaviviruses are enveloped, positive single-stranded RNA viruses. Here, we will focus on three arboviruses from the *Flavivirus* genus in the *Flaviviridae* family, for which birds plays an important role in the virus life cycle: West Nile virus (WNV), Usutu virus (USUV), and Tembusu virus (TMUV). For other avian flaviviruses, refer to Davidson’s review [125].

#### West Nile virus

WNV is a zoonotic arbovirus, nowadays spread on every continent (except Antarctica). The WNV is mostly transmitted by Culex mosquitoes as they take a blood meal. Birds are the principal vertebrate hosts of this virus [126], although WNV infection and disease occur occasionally in humans and mammals (principally horses). WNV was detected in more than 300 bird species [127], with different susceptibility to infection and disease [128]. Passeriformes (incl. *Corvus* genus) and Charadriiformes are the most susceptible birds, developing the most severe (possibly fatal) disease. Interestingly, in Corvidae, WNV was detected in the feather pulp of 77% of the dead birds, almost twice more than in spleen and kidney [129]. In house sparrows, WNV was found to persist at least a month in the skin [130]. Due to their high viremia, these birds play a major role of reservoir by amplifying the virus and being a source of infection for competent mosquitoes [127, 128].

Among farm birds, domestic geese are the most susceptible, showing the highest viremia and possibly developing disease with severe neurological signs [131–135]. High mortality rate (25–40%) was reported in natural infections of goslings in Israel and Canada [133, 136]. Infection of geese by subcutaneous inoculation revealed that among the eight organs tested, the feather pulp was the most positive for WNV genome at 3 dpi [135], indicating that this tissue could be an interesting tissue to sample for diagnostic in birds. WNV RNA was also detected from waterfowl ducks’ samples (organs and swabs) that died in a US commercial farm during an outbreak [137]. In contrast, in experimentally infected ducks, low viremia was observed, with no shedding or symptoms [128, 134]. Such difference may be due to the duck species infected but also to breeding conditions. Although infectable, chickens and turkeys are less susceptible than geese and ducks [134, 138]. Experimentally infected chickens show low and transient viremia (below 10^4^ pfu/mL for 1 to 3 days during the first week of infection) and no symptoms except seroconversion [134, 139]. In consequence, farm birds are not considered as a reservoir/amplifier, their viremia being insufficient to infect mosquitoes, but can serve as sentinels to detect the presence of WNV in mosquitoes through their antibody response [134, 139]. They were often used as such in various geographic areas (for e.g. [140, 141]).

Experimental infections of farm birds are usually performed by subcutaneous needle inoculation or mosquito bite (for examples see [128, 134, 139, 142]). In chickens, viremia is higher after a mosquito bite than needle inoculation [142]. An experimental infection of four chicks showed that Culex mosquitoes inject WNV predominantly extravascularly at the site of feeding (the toe) and little directly into the blood [143]. Phipps reported the presence of viral RNA in skin tissues harvested from chickens inoculated subcutaneously, but not intravenously. Moreover, viral RNA was still detectable in the skin at 3 dpi [144]. Although not investigated yet, it is probable that WNV infects and replicates in skin cells of birds, like it has been demonstrated for mammals. Indeed, WNV was shown to infect keratinocytes at 5 dpi after subcutaneous inoculation in the rear footpads of mice [145]. In human skin explants, WNV infects keratinocytes and dendritic cells, principally in the dermis, but not Langerhans cells [146]. Several questions remain unsolved regarding the difference in birds’ susceptibility, notably the role of the skin in amplifying the virus and of the innate immune response to control the virus at the early stages of infection.

#### Usutu virus

Initially restricted to Africa, USUV emerged in Europe in 1996, and rapidly spread across the continent (reviewed in [147]). Culex mosquitoes are responsible for transmission, similarly to WNV (reviewed in [147]). USUV is highly related to WNV with 76% amino acid identity, complicating serological distinction between the two viruses [126]. USUV infection was recorded in more than 50 bird species of 13 orders, and does not usually induce mortality (reviewed in [148]). However, high mortality was recorded in a few wild bird species, predominantly in Eurasian blackbirds of Passeriformes and also grey owls of Strigiformes [149]. USUV is a zoonotic virus and considered as an emerging threat for humans in Europe [126].

Farms birds are infectable by USUV, but poorly susceptible. Like for WNV, chickens are used as sentinels (e.g., in Italy [150] and in the UK [140]). The low susceptibility of chicken was demonstrated by IV inoculation of USUV in 2-week-old chickens. USUV genome was detected in blood during the first week of infection in all 6 chickens but no clinical signs were observed. More recently, the susceptibility of chickens to four strains of African or European origin was compared after subcutaneous injection of 2-day-old chicks [151]. Microscopic inflammatory lesions were observed in the heart at 5 dpi. Morbidity as well as virus titration from blood, oral swabs, and five organs showed that a chicken line developing low antibody titers is more susceptible than one developing high titers [151]. Feather pulp was not tested in that study. However, in another study, USUV RNA was detected in immature feathers of three inoculated canaries, suggesting that the virus replicates in this tissue [152]. Although not demonstrated, it is probable that USUV infects and is amplified in bird skin, as seen in human and mouse. In human skin explants, USUV infects keratinocytes and Langerhans cells, but not resident dermal dendritic cells [146, 153]. After subcutaneous injection of mice, the viral genome was detected in the skin at the inoculation site, 2 and 5 dpi, but not at distant cutaneous sites [153].

In vitro, USUV replicates in the DF-1 fibroblastic chicken line [151, 154], in a chicken chorioallantoic membrane-derived cell line, and in goose embryonic fibroblasts [155]. Only the last two cell systems show cytopathic effects [152, 156]. Infection of chicken embryonated eggs with high doses of 4 USUV strains of distinct lineages causes embryo death [155]. In that model, USUV is detected in a large number of tissues (including FFs) by immunochemistry, although the presence of infected keratinocytes remains unclear [155].

#### Duck Tembusu

TMUV, identified in 1955, is an emerging flavivirus infecting ducks and chickens (reviewed in [157]). This virus is of economic importance for poultry in Asia, where it induces neurological disease outbreaks. Transmission occurs via Culex mosquitoes [157]. Due to its mode of transmission, it is plausible that, like other Culex-borne flaviruses, TMUV infects skin cells, although this is currently unknown.

### Polyomaviridae and Papillomaviridae

In mammals, notably in humans, Polyomaviruses and Papillomaviruses have a well-known tropism for the skin. In birds, ten virus species have been identified as polyomavirus (reviewed in [158]), all non-oncogenic, with only one naturally infecting domestic farm birds: the goose hemorrhagic polyomavirus [159, 160], which does not induce skin disorders. In contrast, the budgerigar fledgling disease virus (BFDV) causes an acute disease in budgies, resulting in high mortality. In chronically infected adult parrots, BFDV causes mostly integument symptoms, with feather loss and feather abnormalities, especially at re-growth [161]. Interestingly, BFDV does not have a strict host specificity as it can infect chicken and duck cultured cells [161, 162]. The Adelie penguin polyomavirus infection also induces feather loss, the only clinical symptoms [163].

In the last ten years, with the power of next-generation sequencing (metagenomic studies), about 20 new avian papillomaviruses were identified in wild birds, among which duck papillomaviruses [164–168]. Avian papillomaviruses, like mammalian viruses, have a cutaneous or mucosa tropism. Indeed, avian papillomaviruses have been isolated from cutaneous lesions (*Fringella coellebs* papillomavirus 1 and parrot *Psittacus erithacus* papillomavirus 1), healthy skin (for instance Francolinus leucoscepus papillomavirus 1 [169]), and from cloacal swabs/fecal matter (for instance *Pygoscelis adeliae* Papillomavirus 1) [170]. Different types of cutaneous lesions have been reported with these viruses: (i) squamous (cauliflower) papilloma lesions on the foot and lower leg of chaffinches infected by the *Fringella coellebs* papillomavirus 1 [171], (ii) verrucous proliferative lesions on the eyelids and around the beak of African grey parrots infected by the *Psittacus erithacus* papillomavirus 1 [172], and (iii) mesenchymal dermal tumor on the foot of Mallard ducks infected by the *Anas platyrhynchos* papillomavirus 2 [168]. In all cutaneous lesions, virions were detected by TEM [168, 171].

To date, no papillomavirus have been found in chicken and no avian papillomavirus infections have been reported in farms. To this end, several years ago, we explored the skin virome of healthy chickens using cutaneous swabs and could not detect any DNA from papillomaviruses or polyomaviruses [66], like in humans [173–175].

### Parvoviruses

Very little is known on avian parvoviruses and skin interactions although integument disorders have been reported for parvovirus infections, such as the “short beak and dwarfism” syndrome reported in different duck species in several countries [176, 177]. The disease is caused by a goose parvovirus variant and the deformity of the beak appears in the first week of age [176, 178, 179]. Goslings infected at late age show feather loss on the back, neck and wings. The lesions observed in ducks and geese suggest infection of beak epithelial and of feather follicles, but this remains to be demonstrated.

## In vitro avian skin models to study avian virus-skin interactions

Most of the above-mentioned in vitro studies with avian viruses have been performed on monolayer cell culture with non-cutaneous epithelial cells or on chicken embryonic eggs. In particular, several viruses are able to replicate in chicken and duck primary embryo fibroblasts (e.g. fowlpox virus, avian retroviruses, MDV). The advantage of using such cell systems is the ease of preparation and culture. However, some viruses such as MDV present incomplete virion morphogenesis in fibroblasts. In addition, the “behavior” of the cells upon viral infection in vitro varies according to cell types and therefore these models cannot be extrapolated to skin cells. Below we review the skin cell models currently available and briefly discuss how each model could be useful to study the avian viruses-skin interactions. The host response to viral infection in these models is also discussed. Although current models were developed using chickens, some could be easily translated to other bird species.

### Primary cells keratinocytes

Human primary epidermal keratinocytes have been cultivated efficiently since 1975 following procedures innovated by Rheinwald and Green [180]. It is only 30 years later that the serial cultivation of chicken primary keratinocytes (CPKs) was first reported [9]. Basal CPKs are isolated from the body skin of 1-day-old chicks after down removal, and cultivated onto a supporting mouse fibroblast feeder layer (mitomycin-treated 3T3-J2F) with chicken serum. Although it was initially thought that chicken keratinocytes are dependent on the feeder for growth, a recent study reported that CPKs isolated from leg skin can be cultivated without a feeder layer with keratinocyte growth medium [4].

CPKs display a major advantage, which is the potential of differentiation into corneocytes. However, CPKs present also drawbacks such as: (i) a complex and time-consuming protocol to isolate and cultivate them, (ii) a high donor variability, (iii) a short lifetime and a limited potential of serial passaging. In addition, culturing CPKs requires caution to preserve their stem cell properties in order to maintain their proliferative capacity [9]. As of today, only newly hatched chicks and not embryos are utilized to isolate CPKs, although chicken embryos appear to be an easier source of cells and of better societal acceptability. To our knowledge, no serial cultivation of primary keratinocytes has been reported for duck or turkey.

### Keratinocyte cell lines

Obtaining differentiated cell lines remains a challenge in avian biology. Until quite recently (2015), no keratinocyte cell lines were available for avian species. Such model was developed for the first time in our laboratory by differentiating chicken embryonic stem cells (cES) towards a chicken keratinocyte lineage [181]. The differentiation of cES was achieved following induction with BMP4 and ascorbic acid [181]. Three homogeneous populations of cells were obtained: K-cES-K1, -KP2, and -K8 cells. Having keratinocyte cell lines at our disposal to study avian viruses may prove useful considering the limitations encountered with primary CPKs, notably using animals as a source of cells and related ethical issues. We have shown that these keratinocyte cell lines are permissive to the replication of non-pathogenic and pathogenic MDV and lead to the production of cell-associated viral progeny [181]. Nevertheless, despite the presence of all types of virions in cells, no extracellular mature virions have been obtained, suggesting the need of even more sophisticated in vitro models in such context. In addition, these cell lines are most likely models for chicken integument skin and not for hard skin appendages. Although no keratinocyte cell lines have been developed from ducks yet, this appears feasible from duck embryonic stem cells reported in 2010 [182].

Monolayers of keratinocytes, either CPKs or cell lines, are very useful cell systems to compare the replication of various viral strains or recombinant mutants (such as viruses with knocked-out or overexpressed genes). Keratinocytes will also be particularly helpful to identify viral or molecular determinants involved in viral replication and cell-to-cell spread in an appropriate target cell.

### Skin explants cultivation in vitro

Skin explants cultivation in vitro was developed in the early 1960s from chicken embryos, mostly by chicken embryologists (see [183–185]). This system involves maintaining the embryonic chicken skin explants at air–liquid interface on a grid, an insert, or a semi-solid agar support [184, 186]. Another method involves grafting embryonic explants on a chorioallantoic membrane [184]. When embryonic chicken skin explants are cultivated, feather buds can develop [184, 187]. One drawback is the limited time (about 5 days) the skin explant structure and integrity can be maintained in culture. Culture of fowlpox virus [183] and replication of HVT or MDV [57] has been studied in embryonic explants, showing the permissivity of keratinocytes to fowlpox virus and MDV. Skin explants have been harvested mostly from embryos, but they can be theoretically harvested from hatched animals. Such a model has the advantage of having all cells, including immune cells. Moreover, cultivation of the epidermis alone may also be considered to study avian viruses with specific tropism for this layer.

In our opinion, this model has been underused in virology and deserves to be revisited with the new molecular tools of virology (e.g., recombinant viruses), basic knowledge in immunology and cell biology, as well as the new advances in imaging.

### Feather follicle cultivation

Inspired from in vitro hair follicle’s cultivation models [188], we developed in 2022 the first chicken FF in vitro model. After dissecting FFs individually, FFs were immerged in appropriate medium. Such system allows viability of the FFs for 7 days, although the development of feathers is partially impaired [189]. No viral infection with such model has been depicted yet, but this model may help decipher the dynamic steps of feather infection, especially from feather pulp with cell-free viruses, such as influenza virus or retroviruses. The infection could be achieved by injecting the virus directly into the pulp of growing feathers, as Erf and colleagues previously did with antigens in vivo [27]. This model presents the advantage to include all the cells naturally present in feathers and FFs, such as melanocytes, macrophages and possibly Langerhans cells.

### Reconstituted skin equivalent

Very recently, the first avian skin equivalent was reconstructed by using a three-dimensional model [4]. It was obtained by seeding CPKs from chicken leg skin onto a fibroblast-populated collagen matrix before lifting it at an air–liquid interface. Similar systems were developed in mammals since the 1980s (reviewed in [185]). In infectious diseases, they were used in particular to study interactions between the human epidermis and several viruses, such as human herpesvirus type 1 [190, 191], Orf virus, a zoonotic epitheliotropic parapoxvirus [192], or Merkel cell polyomavirus [193].

This model could be interesting to study the interplay between chicken epidermis and several viruses, like Marek’s disease virus or fowlpox virus. This model is particularly interesting to follow a relationship between virus replication and keratinocyte differentiation. This system may also help to decipher the response of keratinocytes to infection or to evaluate the efficiency of antiviral molecules as described for humans [190], even if it is mainly for the purpose of comparative biology and not of bird treatments.

### Potential of skin models for studying the skin immune response

Skin represents the first line of defense against external threats by mechanical/physical (e.g., cornified keratinocytes from stratum corneum tightly joined), chemical (secretion by the epidermis of acids or lipids hostile to pathogens), or cellular/immune (innate or adaptive immune response) barriers. In mammals, keratinocytes participate in the immune defense against pathogens, including viruses, notably through the early innate immune response promoting cutaneous inflammation [194]. Only a few studies have depicted the innate immune response in avian skin in response to skin viral infection. A better characterization of the expression of toll-like receptors and/or mediators of signaling pathways (nucleic acid sensors) in avian skin will help understand the role of skin in viral entry or exit. In addition, as previously mentioned, keratinocytes are naturally rich in lipid droplets. Because lipid droplets were recently shown to play a role in early innate immune response to viral infection in mammalian cells [195], studying the role of these cellular elements in avian keratinocytes in response to viral infection would be of particular interest. All in vitro or ex vivo systems presented above provide a great opportunity to better understand the interplay between skin, viruses, and the immune response.

## Conclusion

The skin and skin appendages, in particular feathers, are a gateway or an excretion site for numerous avian viruses. It is therefore a strategic site to limit infection as well as transmission and/or environment contamination. In addition, feathers, which are easy to collect, are now considered to be a reliable sample for the diagnostic of several avian viral infections (such as Marek’s disease, avian influenza viruses, REV and ALV-J). In the past 10 years, four new skin models were developed in the chicken and could be easily adapted to other avian species. These models open opportunities to study avian virus interactions with natural target cells. They also are particularly important to reduce animal experimentation.

## Abbreviations

ALV: avian leukosis virus
APV: avipoxviruses
BFDV: budgerigar fledgling disease virus
CAV: chicken anemia virus
cES: chicken embryonic stem cells
dpi: days post-infection
DuCV: duck circovirus
ev: endogenous ALV
FF: feather follicle
FFE: feather follicle epithelium
GaHV3: Gallid herpesvirus type 3
HVT: herpesvirus of turkey
GoCV: goose circovirus
ILTV: infectious laryngo-tracheitis virus
MDV: Marek’s disease virus
PBFDV: psittacine beak and feather disease virus
REV: reticuloendotheliosis virus
TEM: transmission electron microscopy
TMUV: Tembusu virus
USUV: Usutu virus
WNV: West Nile virus
